# Distinct Conformations of SARS-CoV-2 Omicron Spike Protein and Its Interaction with ACE2 and Antibody

**DOI:** 10.3390/ijms24043774

**Published:** 2023-02-14

**Authors:** Myeongsang Lee, Marian Major, Huixiao Hong

**Affiliations:** 1 National Center for Toxicological Research, U.S. Food and Drug Administration, Jefferson, AR 72079, USA; 2 Center for Biologics Evaluation and Research, U.S. Food and Drug Administration, Silver Spring, MD 20993, USA

**Keywords:** open-form spike protein, SARS-CoV-2, Omicron, receptor binding domain, angiotensin-converting enzyme 2, antibody

## Abstract

Since November 2021, Omicron has been the dominant severe acute respiratory syndrome coronavirus 2 (SARS-CoV-2) variant that causes the coronavirus disease 2019 (COVID-19) and has continuously impacted human health. Omicron sublineages are still increasing and cause increased transmission and infection rates. The additional 15 mutations on the receptor binding domain (RBD) of Omicron spike proteins change the protein conformation, enabling the Omicron variant to evade neutralizing antibodies. For this reason, many efforts have been made to design new antigenic variants to induce effective antibodies in SARS-CoV-2 vaccine development. However, understanding the different states of Omicron spike proteins with and without external molecules has not yet been addressed. In this review, we analyze the structures of the spike protein in the presence and absence of angiotensin-converting enzyme 2 (ACE2) and antibodies. Compared to previously determined structures for the wildtype spike protein and other variants such as alpha, beta, delta, and gamma, the Omicron spike protein adopts a partially open form. The open-form spike protein with one RBD up is dominant, followed by the open-form spike protein with two RBD up, and the closed-form spike protein with the RBD down. It is suggested that the competition between antibodies and ACE2 induces interactions between adjacent RBDs of the spike protein, which lead to a partially open form of the Omicron spike protein. The comprehensive structural information of Omicron spike proteins could be helpful for the efficient design of vaccines against the Omicron variant.

## 1. Introduction

Severe acute respiratory syndrome coronavirus 2 (SARS-CoV-2) is the hallmark of coronavirus disease 2019 (COVID-19), negatively affecting people’s lives and health since being declared a global pandemic by the World Health Organization (WHO) in March 2020. SARS-CoV-2 belongs to the betacoronavirus genus, and it shares a high percentage of its genomic sequence with the Middle East respiratory syndrome coronavirus (MERS-CoV) and severe acute respiratory syndrome coronavirus (SARS-CoV). Despite the high genome sequence identities of SARS-CoV-2 with MERS-CoV (51.8%) [1] and SARS-CoV (82%) [2,3], the transmissibility and severity of SARS-CoV-2 is much higher than SARS-CoV and MERS-CoV.

The different infection, transmission, and lethality rates between SARS-CoV-2, MERS-CoV and SARS-CoV are mainly caused by their infection processes [4]. Infection is mediated through the spike protein, a ~180 kDa glycoprotein presented on the surface of the viral particles. The spike protein consists of an extracellular domain, a transmembrane anchor region and a cytoplasmic tail [5]. The extracellular domain consists of the S1 receptor binding subunit and the S2 membrane fusion subunit. In the case of SARS-CoV and SARS-CoV-2, the receptor binding domain (RBD) of the spike S1 subunit interacts with the angiotensin-converting enzyme 2 (ACE2) receptors of human respiratory cells, facilitating the entrance of the virus into the host cells [6,7]. MERS-CoV infects cells by binding to the host membrane receptor dipeptidyl peptidase 4 (DPP4), also known as CD26 [8]. It has been demonstrated that the binding affinity between the RBD and ACE2 is much higher for SARS-CoV-2 than for SARS-CoV [9,10]. For instance, Cao et al. showed that the binding affinity between the RBD of SARS-CoV-2 and ACE2 is higher than that between the RBD of SARS-CoV and ACE2 using combined steered molecular dynamics (SMD) simulations and experimental atomic force microscopy [11]. The mechanism by which the spike protein mediates host cell entry has been extensively studied and reviewed elsewhere [5,7,9,12].

Spike proteins are mainly classified as open and closed forms with respect to up and down conformations of the RBD, respectively [7,10,13,14]. It is well documented that an open-form spike protein with an up RBD conformation more easily modulates infection compared to the closed-form spike protein and leads to infection more rapidly than the closed-form spike protein with the RBD down conformation [15,16,17]. Furthermore, the open-form spike protein has a higher probability of binding with antibodies than the closed-form spike protein [15,16]. Although the open-form spike protein with all RBDs in the up conformation is crucial for binding with ACE2 in the corresponding infection process, the structural rearrangement in the subunit S2 of the spike protein impacts the fusion of the virus with host cell membranes [17].

Distinct conformations of the spike proteins of SARS-CoV-2, including wild type and other variants are reported to be associated with the different RBD states [18]. In the trimer structure of the spike protein, three different open and closed forms were observed, which have one, two, and three up RBD conformations. In addition, computational efforts have been made to explore the conformational transition of the SARS-CoV-2 spike protein [19,20,21]. For instance, Gur et al. provided a minimum energy pathway of spike proteins regarded as semi-open spike proteins using molecular dynamics (MD) simulations [19]. Distant residues can modulate the up conformation of spike proteins [21]. Site-specific mutations in the RBD of the spike protein also affect its overall conformation and impact its binding with ACE2 [20]. Therefore, many efforts have been made to explore the distinct conformation states of the spike protein in terms of the number of up RBD conformations to provide extensive structural information of the spike protein for designing vaccines that provide broader protection.

In this article, we review various conformation states of the SARS-CoV-2 spike protein with ACE2 and antibodies. Specifically, we focus on the distinct conformations of the Omicron variant spike protein, which has been the dominant variant among the variants of concern (VOCs) since November 2021. By providing the structural information of distinct conformations of the Omicron spike protein and its interaction features with ACE2 and antibodies, this review could improve the understanding of the Omicron spike protein and provide information crucial for designing new vaccines.

## 2. Structures of Omicron Spike Protein and Its Interactions with hACE2

**Figure 1:**
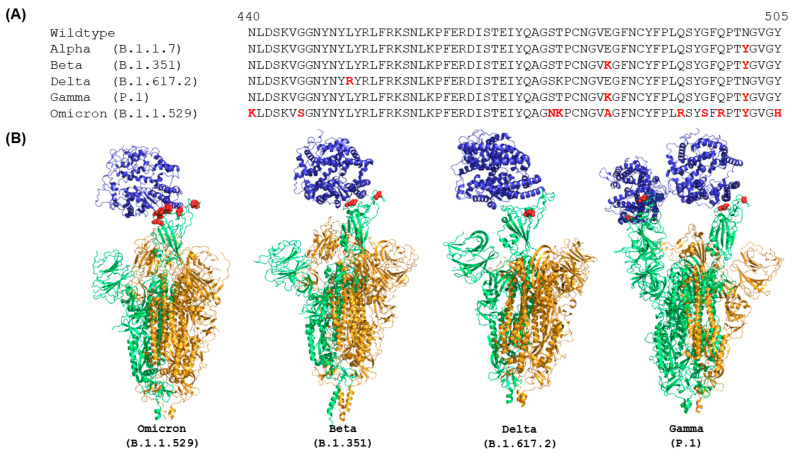
Comparison of RBD sequences (**A**) and ACE2-bound complex structures (**B**) of spike proteins of wild type and VOCs. Sequences are obtained from Yin et al. [16], and the sequence alignment was performed using ClustalW. The mutations in RBD are color coded in red. The human ACE2 are plotted in blue. Green and orange colors represent the chains with RBD in up and down conformations, respectively. The structures plotted in this figure were characterized by EM at the highest resolution and were obtained from PDB (7WPA for Omicron, 7VXD for Beta, 7TEX for Delta, and 7V81 for Gamma).

As of November 2021, multiple VOCs have been reported, including Alpha (B.1.1.7), Beta (B.1.351), Delta (B.1.672.2), Gamma (P.1), and Omicron (BA.1, BA.2, and BA.3) [22,23]. Of these VOCs, Omicron is the currently dominant variant, and over 300 subvariants of Omicron have emerged since November 2021. The remarkable feature of Omicron is that its spike protein has 15 more mutations in the RBD than that of the wildtype spike protein, much more than the other variants, such as Delta, whose spike protein RBD has two mutations. These mutations result in higher binding affinities with ACE2 compared to wild type and other variants [24] (Figure 1). Figures were created using PyMol version 2.0 [25]. Moreover, BA.1, BA.2, and BA.3 are the main subvariants of Omicron reported [26,27]. As of November 2021, BA.2 Omicron had higher transmission rates but less severe symptoms than BA.1 [28]. Compared with BA.1 and BA.2, BA.3 has a low prevalence. These three sublineages have mutations in the RBD of the spike protein, but their mutations are different [29]. For example, BA.2 has unique mutations such as T376A, D405N, and R408S. Therefore, though the reported Omicron variant and its sublineages have homologous sequences, their transmission and symptom features are different. So far, over 600 structures of full-length SARS-CoV-2 spike proteins, including wild type and variants, have been characterized and deposited in the Protein Data Bank (PDB) (https://www.rcsb.org/, accessed on 23 August 2022) [30]. Open- and closed-forms of spike proteins as well as transitional states of the spike protein with 1 or 2 RBD up conformations have been reported. Only the structures of the full-length Omicron spike protein, with and without ACE2, deposited at the PDB website by the end of August 2022 were considered in this review.

Table 1 summarizes structures of trimer spike protein without the ACE2 of the Omicron variant that were determined using EM. Of these 26 structures, 14 consist of closed forms of the Omicron spike protein (RBD is in the down conformation), ten comprise two closed forms and one open form (RBD is in the up conformation), and only two are made from one closed form and two open forms. This observation is well supported by experimental studies that showed RBD-RBD interactions in structures with three closed forms of the Omicron spike protein [31,32,33]. For example, BA.2 Omicron spike proteins were shown as a closed form with three RBD in the down conformations [31,34]. Since the BA.2 Omicron spike protein evolved from BA.1 Omicron, due to some additional mutations in RBD of subunit S1, the BA.2 Omicron spike protein has a different population distribution of open and closed forms compared to the BA.1 Omicron spike protein. The population of spike proteins with one RBD in the up conformation is also higher for Omicron than for other variants, such as Delta, Kappa, and Beta [34,35]. Table 2 lists the structures of the Omicron spike protein with ACE2 that have been resolved by EM and deposited in the PDB website. Of the twelve structures, six have one ACE2 bound with the RBD up conformation, five have two ACE2 molecules bound with the RBD up conformations, and only one has three ACE2 molecules bound with the RBD up conformations. The trimer structures with or without ACE2 demonstrated that the Omicron spike proteins favor trimers with one or two RBD in the up conformation. The trimer with all three RBD in the up conformation may not be stable in most experimental conditions.

From the experimental observations, the binding affinity between the RBD of the spike protein and ACE2 is higher for Omicron than for wildtype [34,35]. Due to the additional mutations in RBD of Omicron spike proteins, more hydrogen bonds are formed between the RBD and ACE2 compared to the wildtype spike protein. Table 3 shows all residue–residue interactions between the hACE2 and RBD up states of Omicron spike proteins. Because the structures were characterized under different conditions, it is essential to comprehensively understand residue–residue interactions. The residue–residue interactions between RBD up conformations of Omicron spike proteins and ACE2 characterized by EM revealed that the additionally mutated residues in RBD, such as H505, R498, S496, R493, and R487, formed hydrogen bonds with ACE2, which lead to a strong binding affinity. These hydrogen bonds were identified in the literature [16,35,43]. The residue–residue interactions, R493-E35, R498-D38, R498-Q42, S496-D38, Y449-D38, Y449-Q42, and Y453-H34, between Omicron spike protein and ACE2 were observed in all Omicron sublineages [16,35,43]. Therefore, these mutated residues in RBDs are crucial for Omicron spike protein interaction with ACE2, which may explain, at least partially, the high transmission of Omicron variants.

The experimentally determined 3D structures displayed different conformations for the RBD of the trimeric spike protein, and thus a different number of ACE2 bound with a trimer of spike protein. However, the driving force behind the structural diversity of the interactions between the Omicron spike protein and ACE2 is not clear. We speculate that this driving force may be associated with the mutation patterns in the Omicron spike protein and deserves further investigation in the future.

## 3. Analysis of Spike Protein with ACE2 between Omicron and Other Variants

It has been determined that the Omicron spike protein has 37 mutations, much more than the spike proteins of the original wildtype strain and other VOCs of SARS-CoV-2 [16]. Importantly, there are many mutations in the RBD compared with wild type, Alpha, Beta, Kappa, and Gamma variants [46]. Alignment of RBD sequences using ClustalW showed that mutations in the RBD of the Omicron spike protein are N440K, G446S, S477N, T478K, S484A, Q493R, G496S, Q498R, N501Y, and Y505H (marked in red in Figure 1A). Furthermore, these mutated residues in the RBD are concentrated on the surface of the Omicron spike protein (marked in red in Figure 1B) that directly involve the interactions with ACE2 and many of the mutations are in the targeting epitopes of therapeutic antibodies in current clinical use [47]. In contrast, other VOCs have much fewer mutations in the RBD of their spike proteins, especially on the ACE2 binding surface (Figure 1B) [48]. Therefore, many mutations in the spike protein, especially on the ACE2 binding surface of RBD and in the targeting epitopes of antibodies, not only change the overall conformation of the spike protein but also impact binding with ACE2 and antibodies.

We examined the interactions between ACE2 and the RBD of spike proteins from different species to understand how the additionally mutated residues in the RBD of the Omicron spike proteins change their interactions with ACE2 [35]. Based on the hydrogen bond measurement between Omicron RBD and hACE2, we summarized the hydrogen bond interactions lost and gained between ACE2 and the RBD of the Omicron spike protein compared with the spike proteins of other VOCs, as shown in Table 4. For example, the Y505H mutation in Omicron generated hydrogen bond interactions with G354 and K353, while the unmutated Y505 formed hydrogen bond interactions with R93 and E37 for Delta and R393 for Beta, respectively. The Q493R mutation of the Omicron spike protein formed a hydrogen bond with H34 of ACE2, the hydrogen bonds between unmutated Q493 with K31 and E35 of ACE2 for Beta spike protein, and the hydrogen bond between unmutated Q493 with K31 of ACE2 for Gamma spike protein were all diminished. In addition, due to the unique mutations in the RBD of the Omicron spike protein, the A475 of the Omicron variant formed hydrogen bonds with Q24 and T27 of ACE2, but the A475 of other VOCs did not form hydrogen bonds with ACE2. On the other hand, K484R mutation resulted in the loss of hydrogen bond formation with ACE2 in the Omicron spike protein, while the unmutated K484 formed hydrogen bonds with K31 and Q75 of ACE2 for Beta and Gamma spike proteins. Overall, the RBD of the Omicron spike protein formed more hydrogen bonds with ACE2 compared with the RBD of spike proteins of other VOCs due to the many mutations in RBD, causing the RBD of the Omicron spike protein to adopt the conformation favoring the formation of many hydrogen bonds with ACE2. The large number of hydrogen bonds between the Omicron spike protein and ACE2 enable the Omicron variants of SARS-CoV-2 to tightly bind with ACE2 so that the virus can quickly enter host cells, leading to a high transmission rate. Our structural analysis provides insights into the structural basis for understanding the infection process and transmission of the currently dominant Omicron variant of SARS-CoV-2 and could help design effective intervention strategies against this variant virus.

The comparative analysis of the experimentally determined 3D structures of spike proteins bound with ACE2 of Omicron and other VOCs revealed the structural basis for the high transmission rate of Omicron variants compared to other VOCs of SARS-CoV-2. However, structures of spike protein bound with ACE2 from different Omicron sublineages, especially the most recently detected sublineages, need to be experimentally determined to provide structures to facilitate the design and development of vaccines that specifically and effectively protect infection with the evolving SARS-CoV-2 variants.

## 4. Structures of Omicron Spike Protein with Distinct Conformation

The currently dominant Omicron sublineages, BA.1 and BA.2, can evade existing vaccine-induced neutralizing antibodies and monoclonal antibodies [29,35,49,50]. Therefore, many efforts have been made to understand BA.1 and BA.2 Omicron spike protein structures with and without antibodies [24,51,52]. Currently, 12 structures of Omicron spike proteins, including BA.1 and BA.2, in the presence of ACE2 sublineages have been determined and reported on the PDB website. A comparative analysis of these spike protein structures has not been conducted. To better understand the infection and transmission of Omicron variants and to provide structural information that could help in the design of effective vaccines, the structural features of the Omicron spike protein complexed with ACE2 were examined.

Before analyzing the difference between BA.1 and BA.2 Omicron spike proteins, the current models of full-length Omicron spike protein with ACE2 as of August 2022 were analyzed (Table 2) to understand the general features of Omicron spike proteins. Of the 12 reported structures of Omicron spike proteins bound with ACE2, only one structure was in a fully open form, where all three RBD are in up conformation. Instead, most structures in the transitional states of the Omicron spike protein were in a partially open form, where one or two RBD are in the up conformation [44]. It is a remarkable feature of the Omicron spike protein that more spike protein chains in the trimer structures complexed with ACE2 have RBD in the up conformation [16,35,53]. For instance, Hong et al. found that the Omicron spike protein with one RBD in the up conformation was the governing model according to their experiments [35]. Compared with other VOCs, the population distribution of the Omicron spike protein with one or two RBDs in the up conformation is higher than that observed for Beta, Delta, and Kappa variants [35,44]. One of the suggested reasons for this phenomenon is that the interaction between adjacent up-conformation and down-conformation RBDs in a trimer of the Omicron spike protein leads to the partial open state of the spike protein that can tightly bind with ACE2 [16,44]. Zhao et al. showed that the interactions between down-conformation RBDs and up-conformation RBDs are caused by the hydrophobic microenvironments in the trimeric structure of the Omicron spike protein. They specifically revealed that the residue mutations S371L, S373P, and S375F increased the hydrophobicity that prevents the additional up-conformation RBDs in the trimeric Omicron spike protein [44]. This RBD-RBD interaction feature has not been observed in the trimeric spike protein of wildtype SARS-CoV-2 due to the movement of the loop of residues 368 to 374 [16]. The strengthened interactions between adjacent RBDs are induced by the mutations in the RBD of Omicron spike protein and cause the high probability of RBDs being present in the down conformation.

**Figure 2:**
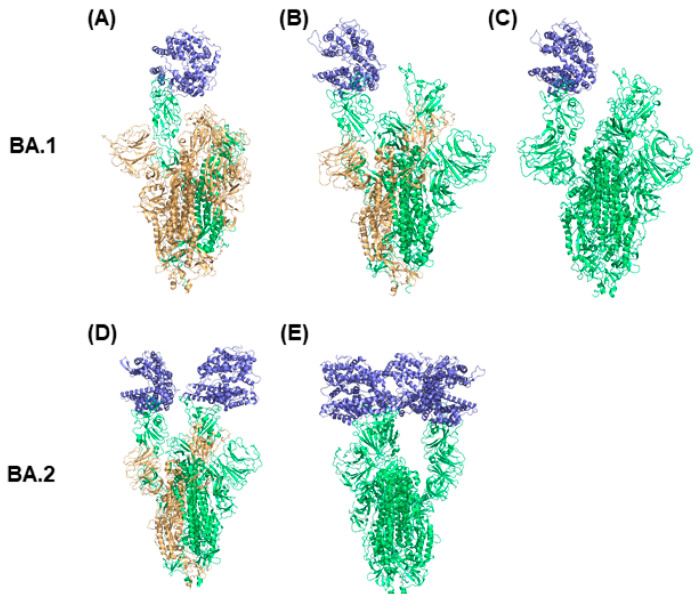
Distinct conformations of Omicron spike protein with ACE2. The conformations (**A**–**C**) are BA.1 Omicron spike protein complexed with ACE2. The conformations (**D**,**E**) are BA.2 Omicron spike protein complexed with ACE2. The spike protein chain with RBD down conformation is marked in orange and the spike protein chain with RBD up conformation is presented in green. ACE2 is colored in blue.

Structural features of Omicron spike proteins can be revealed by examining the difference in structures of BA.1 and BA.2 Omicron spike proteins as shown in Figure 2. Due to the additional mutations on RBD of Omicron spike proteins, BA.1 and BA.2 Omicron spike proteins have different RBD conformations that lead to different binding affinity with ACE2 and infectivity rates for sublineages BA.1 and BA.2 [43]. Thus, it is important to understand the structural differences associated with the preference of ACE2 to bind with BA.1 spike protein compared with BA.2 spike protein. As shown in Figure 2, the BA.2 Omicron spike trimer structures have two or three RBDs in the up conformation, more than those in the BA.1 Omicron spike protein structures, which have one or two RBDs in the up conformation. Because more RBDs in the up conformation make the corresponding structure of the spike protein more open for binding ACE2, the structures of BA.2 Omicron spike protein with more up conformation RBDs may cause higher transmission and infection rates of the BA.2 Omicron sublineage compared to BA.1. The observations from this structure analysis are supported by the findings from Xu et al. that the dissociation constant (*K_D_*) of the BA.2 Omicron spike protein with ACE2 is fivefold and twofold higher than that of wild type spike protein and BA.1 Omicron spike protein with ACE2, respectively [43]. In summary, the BA.1 Omicron spike protein prefers to form a transitional state with one RBD in the up conformation, and the preference of BA.2 Omicron spike protein to having three RBDs in the up conformation is associated with a higher transmission rate compared with the BA.1 sublineage.

New Omicron sublineages are continuing to emerge, including XBB and XBB.1.5, which were detected in December 2022, and data show that vaccinations targeting the spike protein offered some protection (https://www.cdc.gov/mmwr/volumes/72/wr/mm7205e1.htm?s_mm7205e1_w, accessed on 8 September 2022). Our examination of currently available 3D structures showed a distinct conformation of RBD of Omicron spike protein and the conformation difference between Omicron sublineages BA.1 and BA.2. However, whether the observed distinct conformation can be extended to the newly emerging sublineages is to be determined.

## 5. Spike Protein from the Variants with Antibodies

SARS-CoV-2 vaccine development has focused on the induction of neutralizing antibodies. It was reported that the effectiveness of neutralizing antibodies obtained from SARS-CoV-2-infected patients and vaccinated individuals decreased for VOCs, such as B.1.1.7, P.1, and B.1.351 lineages [54]. To improve the binding affinity of antibodies, many efforts have been made involving vaccine design against wildtype and VOCs of SARS-CoV-2. Different types of antibodies have been isolated, and their complex structures with spike proteins have been deposited on the PDB website. Two major forms of structures were included in PDB: isolated RBD of spike proteins complexed with antibodies and full-length spike proteins bound with antibodies. The binding of antibodies or nanobodies to the isolated RBD and the full-length spike proteins are different with respect to the types of antibodies and structural states of the spike protein. In this section, we examine the structures of transitional states of the Omicron spike protein with different numbers of RBDs in the up conformation and their interactions with antibodies, discussing which structure of the Omicron spike protein is favorable for antibody binding.

To date, 39 structures of the Omicron spike protein bound with antibodies have been reported, as summarized in Table 5. With respect to the antibody types and states of the Omicron spike protein, the binding behaviors of antibodies are different. As shown in Table 5, 15 and 12 structures of the Omicron spike protein have one and two RBDs in the up conformations, respectively. Only five and seven structures adopt all three RBDs in the down and up conformations, respectively. An examination of the antibodies bound with the full-length Omicron spike protein found that five structures have only one type of antibody bound with different states of the Omicron spike protein. A representative case is antibody JMB2002, which effectively works against wild type SARS-CoV-2, and the phase 1 clinical trial of JMB2002 is complete [16,55]. The antibody JMB2002 mainly binds the Omicron spike protein with one and two RBDs in the up conformation. For the BA.2.75 sublineage of Omicron, neutralizing antibodies tightly bind the closed form of the spike protein and transitional states of the spike proteins [45]. A study on the Omicron spike protein and 35B5 Fab complex showed that Omicron spike proteins with one and two RBDs in the up conformation are the main targets for 35B5 Fab [56]. Surprisingly, antibody 510A5 can bind various structures of the Omicron spike protein, including fully open and closed forms [53]. Interestingly, although the binding affinity is low, antibody 510A5 locks the down conformation of three RBDs, which potentially contributes to ACE2 binding to the Omicron spike protein. A structural analysis showed a preference of antibodies to the conformation states of partially and fully open forms of the Omicron spike protein.

**Figure 3:**
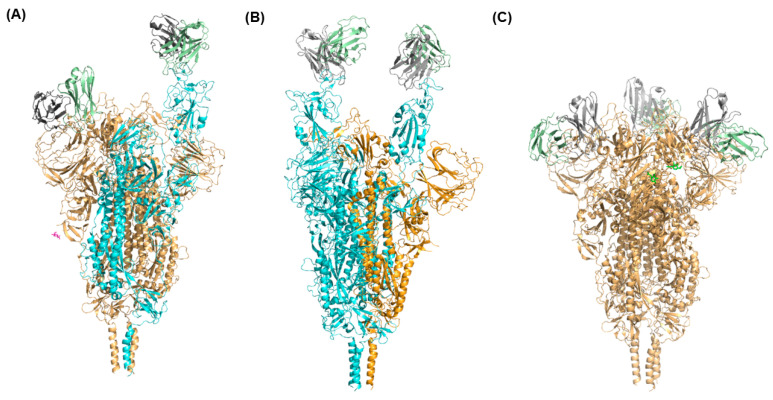
Distinct conformations of the Omicron spike protein complexed with antibodies. The Omicron spike protein with one RBD in up conformation bound with one XGv347 antibody molecule, another RBD in down conformation bound with one XGv347 antibody molecule (**A**), two RBDs in up conformations bound with two XGv347 antibody molecules (**B**), and with all three RBDs in down conformation bound with three XGv347 antibody molecules (**C**). The spike protein with RBD in the up conformation is presented in cyan and the spike protein with RBD in the down conformation is shown in brown.

Some studies suggested that the competition between ACE2 and antibodies affects the conformational states of the Omicron spike protein. For example, Wang et al. characterized structures of different monoclonal antibodies binding with the Omicron spike protein [63]. Different monoclonal antibodies, such as XGv347, XGv282, XGv265, and XGv289 bind to the Omicron spike protein in different ways. Specifically, there are three distinct models for the structure of the spike protein when binding with XGv347: closed-form with all three RBDs in the down conformation, partially open-form with one RBD in the up conformation, and partially open-form with two RBDs in the up conformation. Figure 3 displays the structures of these three distinct models for Omicron spike protein interaction with XGv347 [63]. The binding sites of XGv347 are diverse with respect to the Omicron spike protein states. These distinct models suggest that the competition between antibodies and ACE2 leads to the different states of spike proteins able to bind with antibodies. Antibodies were classified into six classes by evaluating 265 complexes of spike protein bound with the antibodies [65]. The six classes of antibodies can compete with ACE2 to bind to the Omicron spike protein with different states of RBD conformations. The study on complexes of the Omicron spike protein with a Bn03 nanobody also indicated that Bn03-bound RBD interacts with adjacent RBDs of the Omicron spike protein trimer [59]. The down state of RBD caused by Bn03 interacting with the other two RBDs results in a closed-form state of the Omicron spike protein. Therefore, antibodies not only bind to closed-form and transition states of Omicron spike protein but also induce the adjacent RBD-RBD interaction to maintain the states of the Omicron spike protein and to compete with ACE2 to bind the spike protein.

Currently available structures showed that different conformation states of RBD of the Omicron spike protein are caused by antibodies binding. However, antibodies may bind to different sites of the Omicron spike protein. The structural characteristics of the Omicron spike protein bound with antibodies at sites not in the RBD are unknown. They are expected to be elucidated in the future when more structures of spike protein bound with antibodies are experimentally determined.

## 6. Conclusions

The structural features of the Omicron spike protein bound with and without ACE2, as well as those bound with antibodies, were analyzed by reviewing the structures in the PDB. The structural analysis revealed that the trimeric structure of the Omicron spike protein is inclined to form a partially open form that has one or two RBDs in the up conformation. These studies provide insights into the clinical impact of the Omicron variants, with respect to increased transmission rates, showing that the differences in transmission and infection rates among the sublineages of Omicron variants BA.1, BA.2, and BA.3 are associated with the structural characteristics of their spike proteins. Examining the structures of Omicron spike proteins complexed with antibodies indicated that the RBD-RBD interactions within a trimeric spike protein not only lead to the distinct conformations of Omicron spike proteins but also result in antibodies competing with ACE2 to bind the different states of Omicron spike protein. The structural characteristics observed in this review provide valuable information for assisting antibody design and vaccine development that target the Omicron spike protein for future clinical applications. These studies also identify approaches to study further variants that arise as a result of mutations in the SARS-CoV-2 spike protein that may cause higher transmission rates or increased pathogenesis.

## Figures and Tables

**Table 1:** Three-dimensional structures of full-length Omicron spike protein characterized by electron microscopy and the receptor binding domain (RBD) conformation status.

PDB	RBD Form	Resolution	Year	Reference
7TGE	1 down	3.68	2022	[30]
7WK3	1 down	3.4	2022	[30]
7WP9	3 down	2.56	2022	[16]
7TB4	2 down	3.29	2021	[36]
7XNS	3 down	3.48	2022	[31]
7XNR	3 down	3.49	2022	[31]
7XNQ	3 down	3.52	2022	[31]
7XIX	3 down	3.25	2022	[31]
7XIW	2 down	3.62	2022	[31]
7XIY	3 down	3.07	2022	[31]
7WZ1	2 down	3.4	2022	[37]
7QO7	2 down	3.02	2022	[38]
7WK2	3 down	3.1	2022	[35]
7WG7	2 down	4	2022	[34]
7WG6	2 down	3.4	2022	[34]
7TNW	3 down	3.1	2022	[39]
7TO4	2 down	3.4	2022	[39]
7TL1	3 down	3.5	2022	[32]
7TF8	3 down	3.36	2022	[32]
7TL9	2 down	3.5	2022	[32]
7T9J	2 down	2.79	2022	[40]
7UB0	3 down	3.31	2022	[33]
7UB5	3 down	3.35	2022	[33]
7UB6	3 down	3.52	2022	[33]
7THK	3 down	3.11	2022	[33]
7TGW	2 down	3	2022	[41]

**Table 2:** Structures of full-length Omicron spike protein bound with ACE2.

PDB	RBD Form	ACE2	Resolution	Year	Reference
7WGB	2 up	2	3.5	2022	[34]
7WK4	1 up	1	3.69	2022	[35]
7WK5	2 up	1	3.66	2022	[42]
7WPA	1 up	1	2.77	2022	[16]
7WVP	2 up	1	3.7	2022	[35]
7WVQ	3 up	1	4.04	2022	[35]
7XO7	2 up	2	3.38	2022	[43]
7XO8	3 up	3	3.48	2022	[43]
7XCH	2 up	2	3.4	2022	[44]
7Y9Z	1 up	1	2.85	2022	[44]
7YR3	3 up	2	3.52	2022	[45]
7T9K	2 up	2	2.45	2022	[40]

**Table 3:** Residue–residue interactions between Omicron RBD and hACE2.

RBD	hACE2	Reference
Y449	D38, Q42	[35,43]
Y453	H34	[35,43]
L455	D30	[35]
F456	T27, D30, K31	[35]
A475	Q24, T27	[35]
N477	S19	[16,35,43]
F486	M82, Y83	[35]
N487	Q24, Y83	[35]
Y489	T27, F28	[35]
R493	H34	[35]
G494	H34	[43]
R493	E35	[16,35,43]
S494	H34	[35]
S496	K353	[35]
S496	D38	[16,35]
R498	Y41	[35]
R498	Q42	[16,35]
R498	D38	[16,35,43]
T500	Y41, D355, R357	[35]
Y501	Y41, K353, G354, D355	[35]
G502	G354	[35]
H505	K353, G354	[35]

**Table 4:** Hydrogen bond interaction loss and gain between ACE2 and RBD of Omicron spike protein compared to other VOCs.

Lost in	Loss	Gain
Delta	Y505-R93; Y505-E37	H505-G354; H505-K353; R498-D38; R498-Q42; R498; Y41; R493-H34; A475-Q24; A475-T27
Beta	Y505-R393; Q493-K31; Q493-E35; K484-K31; K484-Q75	
Gamma	Q493-K31; K484-K31; K484-Q75	

**Table 5:** Structures of full-length Omicron spike protein complexed with antibodies and nanobodies.

PDB	RBD	Resolution	Antibody Type	Year	Reference
7WJY	2 up	3.24	6m6	2022	[57]
7R40	3 up	2.9	87G7 antibody fab fragment	2022	[58]
7WHJ	1 up	3.27	Bn03, 3 nanobodies	2022	[59]
7WHI	2 up	2.93	Bn03, 4 nanobodies	2022	[59]
7WHK	2 up	3.01	Bn03, 5 nanobodies	2022	[59]
7ZR9	3 up	4	OMI-2 FAB	2022	[59]
7ZR7	3 up	3.7	OMI-42 FAB	2022	[59]
7ZRC	2 up	3.5	OMI-38 FAB	2022	[59]
7XCO	1 up	2.5	S309 fab	2022	[44]
7XOD	2 up	3.27	3 JMB2002 Fab	2022	[43]
7TCC	3 up	3.86	A19-46.1 and B1-182.1	2022	[60]
7TCA	2 up	3.85	antibodies A19-46.1	2022	[60]
7YR1	0 up	3.62	XG2v024	2022	[45]
7YQX	0 up	3.72	S309	2022	[45]
7YQZ	1 up	3.84	S309	2022	[45]
7YQY	2 up	3.74	S309	2022	[45]
7XIC	3 up	3.3	STS165	2022	[61]
8DZI	2 up	3.5	Fab fragment MB.02	2022	[62]
8DZH	1 up	3.2	Fab fragment MB.02	2022	[62]
7WS5	1 up	3.7	510A5 light chain	2022	[53]
7WS0	0 up	3.2	510A5 light chain	2022	[53]
7WS1	1 up	3.1	510A5 light chain	2022	[53]
7WS3	3 up	3.6	510A5 light chain	2022	[53]
7WS4	3 up	3.7	510A5 heavy chain	2022	[53]
7WE7	1 up	3.8	Fab XGv282	2022	[63]
7WE8	1 up	3.5	Fab XGv265	2022	[63]
7WE9	2 up	3.6	Fab XGv289	2022	[63]
7WEA	1 up	3.3	XGv347	2022	[63]
7WEB	2 up	3.7	XGv347	2022	[63]
7WEC	0 up	3.3	XGv347	2022	[63]
7WLZ	1 up	2.98	35B5 Fab	2022	[56]
7WLY	2 up	3.4	35B5 Fab	2022	[56]
7TM0	1 up	3.1	S309, S2L20	2022	[46]
7QTI	1 up	3.04	P2G3, P5C3	2022	[64]
7WK9	1 up	3.48	S3H3 Fab	2022	[35]
7WKA	0 up	3.64	S3H3 Fab	2022	[35]
7WPD	1 up	3.18	JMB2002	2022	[16]
7WPE	1 up	2.69	JMB2002	2022	[16]
7WPF	2 up	2.92	JMB2002	2022	[16]

## Data Availability

Not applicable.
